# Health-promoting lifestyles among suburban women: A critical ethnographic study protocol

**DOI:** 10.1371/journal.pone.0351400

**Published:** 2026-08-04

**Authors:** Faezeh Rastgoo, HamidReza Zendehtalab, Mehrdad Arabestani, Nayyereh Davoudi

**Affiliations:** 1 Student Research Committee, School of Nursing and Midwifery, Mashhad University of Medical Sciences, Mashhad, Iran; 2 Department of Community Health Nursing, School of Nursing and Midwifery, Mashhad University of Medical Sciences, Mashhad, Iran; 3 Department of Anthropology, Faculty of Social Sciences, University of Tehran, Tehran, Iran; 4 Department of Medical-Surgical Nursing, School of Nursing and Midwifery, Mashhad University of Medical Sciences, Mashhad, Iran; The Islamia University of Bahawalpur Pakistan, PAKISTAN

## Abstract

**Introduction:**

Women’s health-promoting lifestyles are a significant global concern for healthcare providers and researchers. International programs, such as the Millennium Development Goals, specifically focus on enhancing women’s health, particularly in marginalized communities. Ethnographic studies offer valuable insights into the cultural contexts and specific needs of these women. Critical ethnography promotes equity and social justice in health by challenging dominant systems and amplifying the voices of marginalized individuals. By gaining a deeper understanding of their challenges, targeted strategies can be developed to improve the health and well-being of marginalized women.

**Methods/design:**

The critical ethnographic approach used in this study is based on the method developed by Carspecken’s 1996, which includes three preliminary (preparation of a list of questions, determination of the required information, researcher value orientation test) and five main stages (Compilation of primary records, Preliminary reconstructive analysis, Dialogical data generation, Description of system relations, System relations, and system relations as an explanation for the findings).

**Discussion:**

This research aims to provide effective solutions for enhancing the health of women living on the outskirts of the city by identifying the lifestyle factors that promote women’s health in these areas and analyzing and critiquing the historical, social, economic, and cultural structures that influence their well-being.

## Introduction

Health-promoting lifestyles have been an international approach and have always been a major concern for health care providers and researchers [1]. Walker et al., in defining the concept of health-promoting lifestyle, stated that a health-promoting lifestyle is a multidimensional pattern of perceptions, feelings, and behaviors that begins with the individual’s own motivation and helps maintain and enhance the individual’s level of health and self-fulfillment. These behaviors include six dimensions: health responsibility, physical activity, nutritional habits, stress management, spiritual growth, and interpersonal relationships [2,3]. This issue has been emphasized in the goals of the World Health Organization’s “Health for All” program. Many of the health problems, such as obesity, cardiovascular diseases, various cancers, and addictions that are seen in most countries today, especially in developed countries, are related to changes in the lifestyle of the people of those societies [4,5]. In this regard, the World Health Organization has announced in the statement of the first Healthy Lifestyle Conference that 60% of global mortality and 80% of mortality in developing countries occur due to the lack of a healthy lifestyle, and by 2030, this probability will reach 75% of global mortality [6]. One of the important factors influencing a health-promoting lifestyle is gender [7]. According to statistics published by the United Nations, women’s health is considered one of the indicators of a country’s development [8]. In many societies, women are disadvantaged by discrimination rooted in sociocultural factors. Some of the sociocultural factors that prevent women from accessing quality health services and achieving the best possible level of health include unequal power relations between men and women, social norms that reduce employment and educational opportunities, an exclusive focus on women’s reproductive roles, and potential or actual experience of physical, sexual, and emotional violence. While poverty is a barrier to health outcomes for both sexes, it places a greater burden on women’s health [9]. Marginalization has been introduced in both the Millennium Development Goals and the United Nations Human Settlements Programme as one of the world’s development challenges, which is influenced by parameters such as economic poverty, type of employment, cultural conflict, illiteracy, ethnic and cultural identity, and lack of control, the effects of which are manifested in the form of some of the acute consequences of urban issues [10]. A general review of past research shows that various studies on the concept of health-promoting lifestyle have been conducted in different cultural contexts and with different research approaches, including quantitative studies [11,12] and qualitative studies based on interviews [13,14]. However, no study was found in which a health-promoting lifestyle was extensively and deeply investigated using participant observation methods in a cultural context. An individual’s lifestyle is always influenced by their culture [15], and in the meantime, cultural behaviors seem to have important consequences for human health, because culture is a social system of knowledge transmission, beliefs, with common practices that manifest differently in groups and societies [16]. Accordingly, conducting ethnographic studies, during which the researcher observes and deeply studies the phenomenon in question in its natural environment, seems appropriate in order to provide rich details about the culture under study and better understand the meanings of behaviors [17]. Critical ethnography is a research methodology that explores how power and domination influence the processes of production, reproduction, and reflection in social life [18]. It is a useful research method in health promotion because it examines the cultural meanings of health, the systemic influences on behaviors, and the relationships between health care providers and patients. It promotes health by empowering marginalized groups and addressing social inequalities. Critical ethnography promotes equity and social justice in health by challenging dominant systems and amplifying marginalized voices [19]. The Carspecken approach, a prominent method in critical ethnography, is used to illuminate how social disparities and structures are perpetuated through routine practices. Carspecken’s method considers critical ethnography as a form of social activism, aiming to uncover hidden systems of dominance and ideologies. This approach also guides data collection and analysis to promote social change and address systemic inequalities [18].

The researchers’ efforts in this study are to discover and explain the meanings, beliefs, internalized values, and behaviors influenced by the health-promoting lifestyle of women living on the outskirts of the city, and to criticize and analyze the historical, social, economic, and cultural structures associated with this culture to gain new insight and knowledge in this regard. They hope that by better recognizing and understanding the needs and problems of marginalized women based on the study results, they will be able to support changes in line with appropriate health planning, such as promoting a healthy lifestyle and helping government organizations and institutions implement appropriate policies to improve the conditions of marginalized women and improve their health, and ultimately move towards ensuring the health of society.

### The main goal

Explaining, describing, and critiquing health-promoting lifestyles in women covered by comprehensive health service centers on the outskirts.

### The project’s practical objectives

Since the findings of this study help to explore culture, they can help develop the knowledge base of policymakers, community health nurses, and health care providers, and pave the way for changes in health planning and the provision of culturally appropriate educational and health services.

### Specific goals of the plan

- Explaining behaviors, beliefs, values, social norms, documents, artifacts, and cultural symbols related to health-promoting lifestyles in women covered by comprehensive health service centers on the outskirts of the city.
- To examine the broader socio-cultural context by critically analyzing the social, economic, and structural factors that shape and influence these health-promoting lifestyles.

### The main question of the research

- What are the behaviors, beliefs, values, social norms, Documentation, artifacts, and cultural symbols related to health-promoting lifestyles among women covered by comprehensive health service centers on the outskirts of the city?
- What are the historical, social, political, economic, and cultural contexts that shape health-promoting lifestyles among women on the outskirts of the city?

## Methods/design

### Ethics approval and consent to participate

The study protocol was approved by the Ethics Committee of Mashhad University of Medical Sciences in accordance with the principles of the Helsinki Declaration and the national ethical guidelines for medical research (ethics code: IR.MUMS.NURSE.REC.1403.008). Written informed consent will be obtained from all participants prior to their inclusion in the study. Participants will be assured that any excerpts from their interviews used in publications will remain anonymous.

### Study status and timeline

Participant recruitment has not yet commenced and is anticipated to begin in December 2025, with completion expected by May 2026. Data collection will continue until July 2026, and data analysis is planned to be completed by September 2026.

### Study design

The present study is based on Carspecken’s critical ethnography method [20]. This method consists of three preliminary stages and five main stages. In the present study, based on the critical ethnography method of Carspecken, three preliminary stages are first carried out before entering the research field.

**The three preliminary stages include:**

A. Preparation of a list of questions
B. Determination of the required information
C. Researcher value orientation test

#### A: Preparation of a list of questions.

At this stage, Carspecken recommends that when the researcher is interested in studying a specific group of people or a specific social place, or a social problem, he or she writes down his or her questions in a list using the brainstorming method. These questions should be general, comprehensive, and flexible; they can change at any stage of qualitative research [20]. Accordingly, in the present study, a list the questions intended by the researchers about the health-promoting lifestyle in women covered by comprehensive health service centers on the outskirts was written:

- How does a health-promoting lifestyle manifest itself in women covered by comprehensive health service centers in the periphery?
- What beliefs, values, and norms make the health-promoting lifestyle of marginalized women this way?
- How does a health-promoting lifestyle of marginalized women manifest itself in documents, manuscripts, records, and tools used in the comprehensive health service center?
- How does a health-promoting lifestyle of marginalized women manifest itself in home visits?
- How do cultural, political, social, economic, and historical structures influence the culture of a health-promoting lifestyle among marginalized women?
- How can the findings be explained and critiqued with relevant social theories?

#### B: Determination of the required information.

In the second preliminary stage, the researcher must specify in another list what information he needs to find the answers to his questions. Usually, this list includes the characteristics of social routines, the characteristics of specific documents, the rules of media products, and the characteristics of the actors who should be interviewed [19,20]. In the present study, the researcher has specified in another list what information he needs to find the answers to his questions, what social routines and specific documents he should observe, and who he should talk to and interview. Accordingly, the compiled list includes:

**Social routines:** creating an electronic health record in the system, providing services such as: education in the field of health promotion and disease prevention and self-care, doctor visits, screening individuals based on the service description (taking blood pressure, diagnosing diabetes using tests and a glucometer, diagnosing breast cancer using the examination method and screening colon cancer using the Faecal Immunochemical Test (FIT), etc.), referrals related to target diseases (communicable and non-communicable), nutritional counseling, psychological counseling, providing active services to the covered population (such as providing complementary services; such as vitamin D, iron tablets, etc.) and following up on people at risk (using health volunteers, sending text messages, calling and following up at home, etc.), daily activities, household chores, relationships with neighbors, ceremonies and celebrations, recreation and entertainment

**Documents:** Forms in the health records of the patients, registration books, signs installed on the walls, and written warnings by officials, and all cultural symbols and artifacts, such as how to dress, customs and traditions, entertainment and hobbies, how to prepare food and cook, etc.

**Rules:** Guidelines and all approved service packages

**Actors:** Physician, health care provider, midwife, and center nurse, environmental health, nutrition expert, psychologist, and women covered by the Comprehensive Health Services Center.

#### C: Researcher value orientation test.

According to Carspecken’s recommendation, at this stage, the researcher should be interviewed by a professional interviewer so that his/her biases and expectations are identified before entering the research field or compiling field notes and organizing the research questions [20]. For this purpose, in the present study, the researcher is qualitatively interviewed by a professional qualitative researcher before starting the fieldwork, then the audio file of this interview is transcribed, and the concepts contained in it are considered in all stages of collecting, analyzing, and writing.

Based on the Carspecken method, five main stages will be implemented in the following order.

**The five main stages of Carspecken’s method are:**

1. Compilation of primary records
2. Preliminary reconstructive analysis
3. Dialogical data generation
4. Description of system relations
5. System relations as explanations of findings

#### 1. Compilation of primary records.

This stage involves conducting fieldwork to gather “first-hand experiences” from the cultural group or community being studied and to uncover their cultural patterns [20]. Fieldwork uses various data collection methods, such as observation and note-taking in the field, interviews and group discussions, and examination of documents and records. Observation in the field of ethnographic studies takes place in four forms: 1 – Complete observer, 2 – participant as observer, 3 – observer as participant, 4 – Complete participant [21]. In the first stage of research, Carspecken recommends that the researcher enter the field as a passive or inactive observer and provide initial documentation from an outside perspective. He believes that the information collected at this stage is monologue because the researcher speaks to himself alone when writing the documents and primary data. At this stage, the researcher does not engage the people under study in any dialogue and simply adopts a third-person position in relation to them and describes them from the point of view of a passive observer [20].

Carspecken has provided specific instructions for participant observation at the level of everyday interactions. For this purpose, Carspecken suggests the observation priority method. Accordingly, he recommends that you first choose one person in the field and record everything that person does and says accurately, then, in the second priority, record everything that another person who is interacting with the first person does and says, and in the third priority, write down everything that happens in the field. Then, every 5 minutes, choose a new person for this observation method. If an event occurs during this observation period that you find important, temporarily take your focus off the subjects so that you do not miss recording this event, but return to the observation priority program after the event is over [20]. To this end, the researcher attempts to describe the date and time, the dominant sense, the smell, the temperature and ventilation, the lighting and illumination of the research site during each participant observation period. Also, during the interactions, the participants’ facial expressions, movements, and body positions, and the tone of their voices are recorded in the form of short notes in the accompanying notebook. At the same time, the researcher’s personal prejudices and analyses will be recorded briefly in the accompanying notebook at an appropriate time. Then, at the end of each participant observation period, and immediately after returning from the research field, all field notes and journal notes will be entered in detail and in a Word file so that the details and sensory events of the interactions are not forgotten [20].

#### 2. Preliminary reconstructive analysis.

This stage involves the initial and inductive analysis of unstructured data. In addition, it focuses on articulating and clarifying cultural and subjective elements, which are often implicit in nature. The analysis here is reconstructive and aims to articulate cultural themes and systemic factors that are usually not visible and not directly experienced by the participants involved in the communication.

This stage includes: low-level coding, initial meaning reconstruction, horizon analysis [20].

I. Low-level coding. This stage is done on primary records and requires minimal abstraction [22].
II. Initial meaning reconstruction: To reconstruct the initial meanings, the researcher must select multiple segments of the primary records simultaneously with the low-level coding. The selected components should represent behavioral patterns, reflect pattern anomalies, or reveal underlying norms of routine events. Next, the researcher has to copy these pieces into a separate Word file, read them line by line, and articulate the hidden meanings they think are the basis of the interaction by writing “meaning fields”. The meaning field is a range of possibilities, and the purpose of writing meaning fields is to use more words for the description of the observed actions in such a way that if the actor had tried to express all the meanings of the action verbally (not through vocal tone, posture, gesture, facial expression, timing, and prosodic forms), they would have used these sentences [20,23].
III. Horizon analysis: Horizon analysis is about the fact that we cannot comprehend ideas in the world without simultaneously understanding the horizon from which they arise. Analysis of the horizon gives us clues about what is socially acceptable in the situation under study and completes our perceptions of the cultural environment [24]. In the process of articulating validity claims in horizon analysis, subjective, objective, normative, value, and identity claims in meaningful acts are analyzed at the horizontal level. Carspecken argues that these claims are also pursued in a continuum from the highly foregrounded to the deeply backgrounded. In the process of horizon analysis, Carspecken calls this level of analysis vertical analysis. In this argument, he deals with cultural structures and their various layers. According to him, some cultural categories, beliefs, and themes are located in the surface layers of culture that people are aware of; nevertheless, culture also has deeper layers and structures. He considers vertical analysis a tool through which the researcher can identify the deep layers of culture [19].

According to Carspecken, the coding process should not be closed until the end of the third stage, because the third stage emphasizes conversational data and confirms the evidence from observations, so the interviews should also be coded [20]. In the present study, the coding process will be carried out according to Carspecken’s guidelines. First, low-level coding is carried out on the primary records, while simultaneously identifying meaningful segments and writing meaning fields for them using the obtained codes. Horizon analysis is then conducted for each of these meaning fields. From this analysis, high-level codes are reconstructed. In the next step, the primary records are re-examined to find codes or initial phrases corresponding to each high-level code, using horizon analysis. This process creates a hierarchical structure of low-level and high-level codes that can later be merged to form larger categories and create main categories [19,20,23].

#### 3. Dialogical data generation.

In the third stage of critical ethnography using Carspecken’s method, interviews will be used to collect data. Carspecken believes that the ideal qualitative interview should be semi-structured and that the researcher should prepare an interview protocol that allows him or her maximum flexibility during the interview process [20]. The protocol introduced by Carspecken consists of four parts: the topic domain, lead-off questions, covert categories, and possible follow-up questions. The general scope of the interview topics is specified in the topic domain. Then, the lead-off questions are designed to open up these domains. Carspecken believes that the best lead-off question is one that asks about an event that the participant was a part of and the researcher witnessed, which should be very concrete and clear and not abstract. Then, as the participant describes the event, the researcher attempts to elicit, with appropriate questions, the underlying beliefs, values, beliefs, and feelings involved in that event. Carspecken recommends that for each topic area, a list of items that the researcher would like the participant to mention during the interview, but does not want to ask about explicitly, be written down. These are called covert categories. In this way, the researcher organizes these items in his or her mind so that he or she can refer to them during the interview and not forget anything important. Therefore, when a topic domain opens up, the interviewer must skillfully guide the participant’s conversation through the lead-off questions. Finally, the researcher should write down possible follow-up questions for each topic area. Carspecken recommends that in cases where the interview is very far from the time of observation or the researcher wishes to simply interview a participant, it is better to ask questions about events that occur routinely in the context [20]. During participant observation, the researcher may need to interview or talk to participants to answer his or her questions and interpret what he or she has observed. In fact, interviews are used to clarify, validate observations, and guide further observation [19].

Accordingly, in the present study, interviews and conversations about observed events and interactions will be conducted by the researcher to uncover the meanings, beliefs, values, and convictions that underlie behavior. In addition, interviews will be conducted using an interview guide suggested by Carspecken to help answer the research questions. Interviews will be conducted in-depth, with due regard for psychological safety, privacy, and confidentiality of information, and will be recorded with the consent of the participants. The time and place of the interview will be determined by the participants themselves. Accordingly, in the present study, the researcher attempts to collect appropriate conversational data during participatory observation and, if necessary, uses interviews to complete concepts and questions. To this end, the researcher develops an interview protocol based on the proposed protocol of Carspecken for conducting formal interviews with key informants, revises it based on the opinions of experts, and then uses it in formal interviews. For example:

**Topic domain:** Health-promoting lifestyle.

**Lead-off question:** How do you evaluate your health status? What do you do to maintain your health? What factors can help improve your health? What makes you do or not do behaviors that increase your health? Please tell us about the health-promoting behaviors you have experienced.

**Covert categories:**

- Belief: What do you think about this? What makes you think this way?
- Acceptance and belief: Do you accept or not accept it?
- Feeling: How did you feel?
- Values: Why should it be this way or not?
- Expectations: What do you expect?

**Possible follow-up questions:**

- What do you mean? Please explain more.
- Can you give an example?
- What else do you remember about this?

Furthermore, Carspecken suggests that documents should also be reviewed in the study [20]. In the present study, the documents and records available in the Comprehensive Health Services Center, various documents and records related to the research questions, including minutes, policies, guidelines, health care provider reports, educational materials (brochures, pamphlets, conferences), and all cultural symbols in the women’s living areas, such as; how to dress, customs and traditions, recreation and entertainment, how to prepare food and cook, etc., will be reviewed to find examples of health-promoting lifestyle culture.

#### 4. Description of system relations.

The purpose of the fourth stage of Carspecken’s method is to describe the relationship of the system with a broader context. The researcher determines the integrity of the system by testing several sites related to the study site and experiencing the cultural reconstructions already developed. The researcher asks about the origin of the emerging cultural themes, and the answer to this question will lead them to related places and situations. Accordingly, to analyze social structures, existing inequalities, injustices, and cultural ideologies based on the principles of critical theory [19], the researcher can ask the following questions:

1. Why did these particular cultural themes emerge? This question is usually answered through historical considerations and the study of the socio-economic and political environments in which people live and work.
2. How do cultural forms acquired in one social site relate to other cultural forms in other social sites? This question is answered through searching other social sites that are frequently referenced and repeated by study participants [20]. In addition, media and other cultural goods are important factors to consider [19], because a particular cultural product can influence the relationships and discourse within the study site and therefore needs to be analyzed carefully [20].
3. What functions do the consequences of action fostered by cultural forms serve in larger social networks? In this area, class and gender reproductions have often been emphasized by critical researchers [19].

In the present study, the proposed guidelines for analyzing systemic relations, as explained above, will be used. Accordingly, in the first stage of systemic relations, the findings of the present study are presented within the framework of analyzing historical, social, economic, and political structures. Then, in the second stage, the cultural forms obtained in the current research field are compared with the cultural forms of other social places, and the impact of media and cultural goods on the concepts obtained from the research field is examined and critiqued. In the final stage, the impact of the consequences of the actions and cultural forms produced in the research field on larger social networks is addressed.

#### 5. System relations as an explanation for the findings.

In the fifth stage of Carspecken’s method, the findings are analyzed and interpreted in the light of general social theories to explain what has been discovered in stages 1–4 with the help of these theories. Moreover, at this stage, the macro-sociological theories are challenged, changed, or modified [19,23].

### Research field

The research field in ethnography is the place where the phenomenon occurs; in other words, the research is conducted in a place where the people in question live and their experiences occur [25], and it is the research question that determines the field and participants of the research [26]. Data collection will be conducted by the primary researcher, who will engage in prolonged fieldwork across multiple settings (e.g., comprehensive health service center, participants’ homes, schools, hair salons, bakeries, butchers, herbal shops, shopping malls, weekly markets, buses, parks, social events, religious gatherings, charity and women’s parties, and informal group meetings). This approach facilitates in-depth understanding and trust-building with participants, which are essential components of ethnographic research.

### Participants

Participants in ethnography are literally sources of information and teachers for the ethnographer, and in ethnography, the ethnographer uses the language of the informants to describe the culture [26]. Accordingly, in the present study, participants are all individuals present in the research field who play a role in the lifestyle promotion of women on the outskirts of the city directly or indirectly and experience this phenomenon (such as doctors, health care workers, midwives and center nurses, environmental health workers, nutritionists, psychologists, etc.), but women covered by comprehensive health service centers on the outskirts of Mashhad are considered the key informants. These individuals will be included in the study if they are willing to participate in the research.

### Data collection methods

In the present study, the researcher attempts to collect data related to the research questions by being present in the research field for a long time (about a year) and gaining the trust of the participants. Given that the basis of data collection in ethnography is triangulation [27], in the present study, the researcher will collect ethnographic data during fieldwork to investigate the phenomenon of interest, which is a health-promoting lifestyle, and based on the project manager’s instructions, will use participatory observation methods, conversational data and interviews, and document review to collect data, which is fully explained in the methodology section.

### Data analysis

In the present study, after low-level coding, selecting meaningful segments, and writing meaning fields, horizon analysis will be carried out on the meaning fields. In this way, the possible meanings of actions will be inferred according to the horizontal levels, and based on the analysis, the horizon of high-level codes will be inferred. Then, the high-level codes with similar meanings will be merged, and in this way, the intermediate categories will be inferred. After that, the main categories will emerge from the merger of the intermediate categories. In the second phase of the present study, the description of systemic relationships is carried out, that is, the findings from the study are analyzed in a systematic way. At this stage, the researcher must determine what and where the origins of the cultural themes emerging from the field are. And the answer to this question will lead her to relevant places and situations. In the present study, the researcher must discover important systemic relations, and to answer this question, it may be necessary to conduct fieldwork in city center health centers or field visits. Also, to identify the origins of the cultural themes emerging from the field, the researcher must fully identify and analyze the historical, political, economic, social, and cultural origins of the findings. In fact, the goal of this stage is to begin an analysis that is not limited to the interpretations or meanings of individuals’ narratives, but allows explicit links and connections to be established with broader sociological events that affect the field of study [28].

The end phase in the present study is systemic relationships as an explanation for the findings, in which the researcher must relate the actual evidence to theoretical concepts and explain the micro findings related to stages one to four using macro theories related to the findings.

## Discussion

Healthy lifestyles are widely recognized as a key factor in promoting health and preventing disease, especially among women, especially in marginalized areas [29,30]. Due to economic and social conditions, women in these areas often face challenges such as limited access to health services, inadequate health information, lack of access to appropriate exercise facilities, high prices of healthy food, and lack of social support, which can negatively affect their ability to achieve a healthy lifestyle [31,32]. Cultural and social factors also play a significant role in determining women’s lifestyles [31]. Social beliefs and attitudes about the role of women in society can influence their health behaviors, especially in the areas of nutrition and physical activity [33]. For example, in some cultures, women may face family and social barriers to dedicating enough time to exercise or choosing healthy foods. Critical ethnography helps researchers identify these barriers and analyze the problems with a deeper understanding. It also provides an opportunity to gain a deeper understanding of women’s social and cultural responses to their social and economic environments, in addition to collecting qualitative data.

Using the findings of this study, stages can be taken to increase women’s awareness and determine the priorities for providing services to women covered by comprehensive health services on the outskirts of the city. Also, by determining the existing challenges, it is possible to help reduce existing barriers by creating social and economic infrastructures, such as creating free sports spaces and access to healthy foods.

Finally, this study shows the importance of adopting a comprehensive approach that is appropriate to the cultural and social needs of women in health planning. It is appropriate for policymakers and decision-makers to pay serious attention to this issue and, by utilizing the results of such studies, take action to improve the quality of life of marginalized women.

### Plain language summary

Women’s health-promoting lifestyles are a global issue of importance to healthcare providers and researchers. Ethnographic studies help us understand their cultural needs and challenges, which leads to better health strategies. Critical ethnography is a useful research method in health promotion because it examines the cultural meanings of health, the systemic influences on behaviors, and the relationships between health care providers and patients.

This study uses a critical ethnographic approach based on Carspecken’s 1996 methodology, which includes three preliminaries (preparation of a list of questions, determination of the required information by the researcher, and researcher value orientation test) and five main stages (compilation of primary records, preliminary reconstructive analysis, dialogical data generation, description of system relations, and system relations as an explanation for the findings).
